# Complexity synchronization in living matter: a mini review

**DOI:** 10.3389/fnetp.2024.1379892

**Published:** 2024-05-20

**Authors:** Bruce J. West

**Affiliations:** ^1^ Department of Research and Innovation, North Carolina State University, Raleigh, NC, United States; ^2^ Center for Nonlinear Sciences, University of North Texas, Denton, TX, United States

**Keywords:** fractal time, complexity synchronization (CS), complexity measure, multifractal dimension, triad of EEG, ECG and resperation

## Abstract

Fractal time series have been argued to be ubiquitous in human physiology and some of the implications of that ubiquity are quite remarkable. One consequence of the omnipresent fractality is complexity synchronization (CS) observed in the interactions among simultaneously recorded physiologic time series discussed herein. This new kind of synchronization has been revealed in the interaction triad of organ-networks (ONs) consisting of the mutually interacting time series generated by the brain (electroencephalograms, EEGs), heart (electrocardiograms, ECGs), and lungs (Respiration). The scaled time series from each member of the triad look nothing like one another and yet they bear a deeply recorded synchronization invisible to the naked eye. The theory of scaling statistics is used to explain the source of the CS observed in the information exchange among these multifractal time series. The multifractal dimension (MFD) of each time series is a measure of the time-dependent complexity of that time series, and it is the matching of the MFD time series that provides the synchronization referred to as CS. The CS is one manifestation of the hypothesis given by a “Law of Multifractal Dimension Synchronization” (LMFDS) which is supported by data. Therefore, the review aspects of this paper are chosen to make the extended range of the LMFDS hypothesis sufficiently reasonable to warrant further empirical testing.

## 1 Introduction

In a recent series of papers Mahmoodi et al. (Mahmoodi et al., 2023a; Mahmoodi et al., 2023b) and West et al. (West et al., 2023) have used the scaling behavior of dynamically generated time series to hypothesize the existence of a new form of synchronization, called complexity synchronization (CS), having to do with the optimally efficient exchange of information between and among the organ-networks (ONs) within the human body viewed as a network-of-ONs (NoONs). This complexity synchronization (CS) involves the matching of the multifractal dimensions (MFDs) for time series and was identified by processing a 64-channel electroencephalogram (EEG) of the human brain with each channel treated as a distinct network, while the brain continuously interacts with the respiratory and cardiovascular networks. The complexity that is being synchronized is measured by the inverse power law (IPL) index of the power spectral density of the MFD time series. The essence of synchronization has to do with adjusting periodic rhythms arising from an interaction in which one network influences another through the exchange of information. Even with this apparently simple-sounding definition of synchronization we find it necessary to distinguish among a few different kinds of synchronization to clarify the difference between the old forms and the new ones considered herein.

The first and subsequent kinds of traditional synchronization between interacting time series is extensively treated in the excellent text (Pikovsky et al., 2001) which provides a brief history of the universality of the concept of synchrony pointing out that in 1665 Huygen’s discovered the phenomenon of *mutual synchronization* between two pendula sharing a common mount (Huygens Hugenii et al., 1986); remarking that two centuries later Strutt (Lord Rayleigh) described an extension of mutual sychronization occurring when two similar but separate organ pipes are sounded together to produce *quenching* which is the suppression of oscillations of the sound of the coupled systems (Rayleigh, 1945); and closing their all too short chronicle observing that with the birth of radio engineering Eccles and Vincent applied for a patent (Eccles and Vincent, 1920) for a device consisting of two coupled generators having slightly different frequencies establishing that the coupled system vibrates with a single frequency. They end their historical vignettes in the 1920s after which time a substantial variety of synchony types began to appear in the scentific literature and which they discuss in their text.

Collective synchronization (van Santen, 2019) is seen all around us, from the flocking behavior of birds (Hemelrijk and Hildenbrandt, 2015) to the herding movement of crowds and vehicles (Vicsek, 2001) and the well-documented avalanches of neurons within the brain (Beggs and Plenz, 2003; Buzsáki, 2006) and are all engagingly described by one of the pioneers in the field (S Strogatz, 2003). Strogatz pointed out that there are over a half-dozen different types of synchronizations depending on the phenomena being described. For those that do not have mathematics as a second language the first half of the book by Pikovsky et al. (Pikovsky et al., 2001) is equation-free and devoted to the qualitative description of the forms of synchrony discussed in mathematical detail subsequently therein.

Synchronization may be understood to be an adjustment of rhythms of oscillating objects due to their weak mutual interactions but like most definitions it is necessary to further refine the definition by means of definitions of the technical terms. Pikovsky et al. (Pikovsky et al., 2001) break down the above definition by detailing the meaning of “oscillation object”, the notion of “rhythm”, the concept of “interaction of oscillating systems” and the technical meaning of “adjustment of rhythms” in ten pages of text. I cannot improve on this and therefore I am forced to assume the reader has a working familiarity with each of these terms but may not be familiar with the wide range of phenomena that have been explained using the notion of synchronization. We briefly review the dominant characteristics of synchronization to be clear on what is different and what is the same regarding the newly defined synchronization mechanism.

Consider two time series *X(t)* and *Y(t)* from two different sources and we observe that both oscillate in complete harmony which can only be associated with synchronization if: (Pikovsky et al., 2001):

1) It is established that when not interacting that both systems generate their own rhythms.
2) The systems adjust their rhythms by weakly interacting with one another.
3) The rhythm adjustments fall within a given range of mismatch.

Synchronization can also be generated by an external force, by ensembles of oscillators and oscillatory media, through phase synchronization of chaotic systems and complete synchronization are all discussed by Pikovsky et al.

## 2 Not your usual synchronization

Unlike the familiar forms of synchronization of two time series, we are here concerned with another kind of synchronization which enables us to look behind the curtain and observe the wizard at work on his dynamic elements within complex networks and witness their collective interactions. Let me emphasize here that this is not the kind of synchrony that we all too briefly reviewed in the previous section nor is CS to be found in any of the chapters of the comprehensive introductions to the rhythms within complexity science (Bak, 1996; Peletier et al., 2019; S Strogatz, 2003). Note that CS was discovered two decades after Strogatz’s remarkable book raised the overall awareness of the science, technology, engineering, and mathematics (STEM) community to the singular importance of synchronization research in the understanding of the behavior of complex collective adaptive systems (Stefanovska et al., 2003; S Strogatz, 2003; Turalska et al., 2011) as well as that of fractals in the life sciences (Liebovitch, 1998). The discoveries are ongoing from the fractal random growth of interfaces (Failla et al., 2004) to the pumping of playground swings (Hirata et al., 2023) to the modification of fundamental concepts in statistical physics (Aquino et al., 2010; Aquino et al., 2011).

Modern synchronization has been shown to be the mechanism needed to coordinate activities among events in any complex, multi-level, multi-element dynamic network living or not (Miller and Page, 2007). However, as an ON’s complexity changes so does the synchronization of its dynamic interactions with other ONs which makes the living network significantly more difficult to characterize than the non-living (West et al., 2020). The coupling of these two kinds of change is nowhere more apparent than in the remarkably complex ON structure of the human body and the necessity to coordinate activities across widely different time scales from the microscopic time scales of the neural ONs within the brain to the mesoscopic time scales of the cardiac ON to the macroscopic time scales of respiratory ON and circadian NoONs rhythms.

The complexity (scaling) of EEG time series data (64-channel electroencephalogram) were shown to be multifractal as were the respiratory and cardiovascular time series in addition to which the multifractal dimension (MFD) scaling of the three kinds of simultaneously measured datasets were shown to be in synchronization with one another (Mahmoodi et al., 2023a; Mahmoodi et al., 2023b; West et al., 2023). The MFDs of these time series have also been identified using pairwise correlations between time series to identify an appropriate mechanism (Bartsch and Ivanov, 2014). The change in the MFD of the time series indicates the changing complexity of the ONs as various physiological functions are performed. In the jargon of network science for two interacting ONs, the more complex network is the driver (sender of information) and the less complex network is the driven (receiver of information) but these roles of various EEG channels change with the functions being performed as do the respiration and cardiac time series (Ivanov et al., 1999; Stefanovska et al., 2003) and they each change in time as well depending on the function being performed.

The major distinction between the present work and such theoretical works as those of Plamen Ivanov and his group (Ivanov et al., 2016) is that they are working towards understanding, “complexity ... compounded by various couplings and feedback interactions among different systems, the nature of which is not understood” from a network physiology and/or network medicine theory perspective. We on the other hand, are working towards the same end from the other side which is to say through understanding the generic empirical properties that have only recently been uncovered and precisely how such phenomena as CS can and do constrain theory development is only now being glimpsed (Mahmoodi et al., 2023c; West et al., 2023).

Information is readily exchanged within overlapping memory areas of the heterogeneous human brain. At any point in time a given region of the brain (a channel is treated as an ON) can receive information (a brain channel acting as a receiver-ON) from a sensor-ON and is driven by this input information to locally process the received signal and subsequently transmits (the same brain channel changing to sender mode) the processed signal to an appropriate physiological-ON for action, depending on their function and instantaneous relative MFDs. This hierarchy of complexity is subsequently revealed by the way in which the MFD nature of the interacting ONs exchange information with one another over time. The relative width of the MFD spectra of an ON’s time series at the time of an interaction determines which is the sender (greater width) and which the receiver (smaller width) (Braginton, 2003; West and Grigolini, 2021).

Xiao et al. (Xiao et al., 2021) constructed what they termed a node-based multifractal analysis (NMFA) which is a theoretical framework revealing the generating rules and quantification of the scale-dependent topology of dynamic complex networks. They showed that the NMFA yields precisely the singularity spectrum *f*(*α*) obtained using the thermodynamic arguments (Feder, 1988; Meakin, 1998). The theory of (Xiao et al., 2021) supports the intuitive statements made regarding the behavior of the empirical ON-generated MFD time series based on the empirical evidence for the complexity matching principle (West et al., 2008; Delignières et al., 2016; Bohara et al., 2017), but now has a direct underlying theoretical argument.

The depth of our understanding of synchrony stands in sharp contrast to the shallowness of our collective insight into the mystery that is complexity science whose fundamental problems have gained preeminence in science over the last decade paralleling the sharp rise in the application of Network Science to our understanding of collective behavior in medicine in general and in physiology in particular (West, 2014; Ivanov et al., 2016). One might say that the modern view of complexity began with the acknowledgement that a new branch of science had formed around the behavior of a many-degree-of-freedom system governed by the emergent dynamics of spontaneous self-organized criticality (SOC) (Bak, 1996). Moreover, both bottom-up resilience and top-down vulnerability arise from emergent processes captured by spontaneous self-organized temporal criticality (SOTC) (Mahmoodi et al., 2018). The term criticality is used herein to denote the dynamic condition corresponding to the spontaneous onset of phase transitions generated by the self-induced adoption of a critical value of a control parameter by the internal dynamics.

**FIGURE 1:**
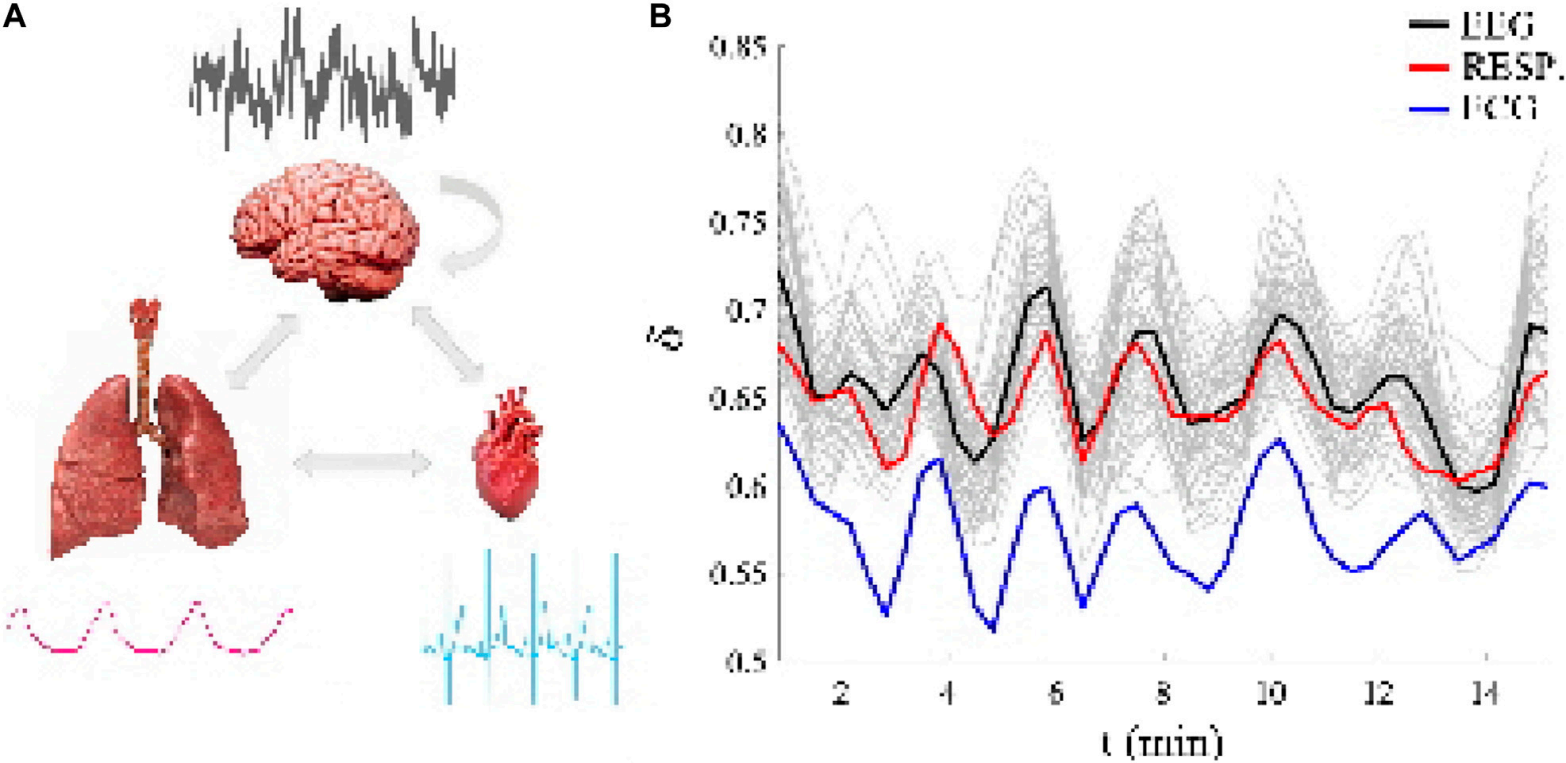
**(A)**: The three ONs whose fractal time series outputs are simultaneously measured and representative 10 s samples of each time series are shown for each ON drawing to emphasize how different the time series are in both time scales and statistical structures. **(B)**: Each of the 66 channels are different fractal time series having a unique multi-fractal dimension as determined by the time-dependent scaling parameter *δ*_*j*_(*t*), *j* = 1, 2, .., 66, over a 15 min time interval. The black line segment is the average over the 64 channels of the EEG multi-fractal dimension time series (the 64 light gray line segments in the background are the EEG channel multifractal dimensions in the brain), the red line segment is the multi-fractal dimension of the respiration fractal time series and the blue line segment is the multi-fractal dimension of the electrocardiogram (ECG) fractal time series. Note the similarity of the individual multi-fractal dimensions of the three kinds of time series, particularly their quasi-periodic behavior. Adapted from (West et al., 2023) with permission.

In Section 2.2 and SM#2 we review the scaling properties of MFD time series to make clear the implications of the DEA data processing results shown in Figure 1. We mention that here because the scaling index gives a direct measure of the multifractal dimension of the time series which for the crucial event time series (CETS) discussed in Section 2.3 has diverging second moments thereby making all processing techniques suspect that are based on the time series being processed having a finite second moment.

### 2.1 Basis of complexity synchronization (CS)

As pointed out by West et al. (West et al., 2023) the science motif of CS is based on scaling determined by the 1/f-variability of complex dynamic ONs and the need for a NoONs to transport information internally during intra-ON dynamics and externally exchange information which is the interaction among ONs during inter-network dynamics among the members of the NoONs. The term 1/f-variability is used throughout rather than the more familiar term 1/f-noise because the author finds the latter term to be directed toward random fluctuations that carry no useful information whereas the former term addresses time series with inverse power law (IPL) statistics. The IPL index in the latter case provides a working definition of complexity that being the multifractal dimension (MFD) and is used as a measure of complexity of the crucial event time series (CETS) studied herein. The quantification of the complexity adopted herein is the MFD which is shown to be a measure of the complexity content of CETS that constitutes the signal generated by an ON. Moreover, the difference in the MFDs of two interacting ONs (such as the cardiovascular and respiratory) within a NoON (a human body) quantifies the relative complexity between them as they interact within a NoON (West and Grigolini, 2021) as well as the higher-order interactions between NoONs (two or more human bodies), e.g., see (Kello et al., 2007; West et al., 2008; Almurad et al., 2018).

The mathematician Norbert Wiener observed (Wiener, 1948) that in the interaction of two physical networks heat is transferred from the hot to the cold system when the entropy of the two systems is comparable. He went on to conjecture that when the hotter system is relatively low in information and the cooler system is relatively high in information the force generated by the information gradient between the two systems may be stronger than the mechanical force generated by the energy gradient between them and acts in the opposite direction. Thus, resulting in the hotter system being controlled by the cooler system which at first glance suggests a contradiction to the Second Law of Thermodynamics. Wiener goes on to argue that this second force, the entropy force, is completely consistent with the second law. The “entropy force” has been used to explain the elasticity of rubber (Neumann, 1977) and the physical properties of colloids (Damasceno et al., 2012) among many other things, see, for example, (Sharp, 2012). Herein we replace the nomenclature “entropy force” with “information force” and concentrate our remarks on those systems dominated by such information forces (West, 2016).

Fractal statistics are ubiquitous in the determination of physiological fluctuations in that the waiting-time distribution of the time intervals between successive beats of the human heart (Ivanov et al., 1999; Allegrini et al., 2002; Humeau et al., 2010), between breaths in respiration (Bohara et al., 2017), and in the critical dynamics of the brain (Kello et al., 2007; Allegrini et al., 2009), are all IPL in time and result from intra-ON interactions or paraphrasing Buzsaki (Buzsáki, 2006) on the fractal nature of the human brain: the human brain’s web of influence consists of a multilevel, self-similar organization which a physicist would call a fractal network of ONs. Similar findings regarding the time intervals between events are documented for the information exchanged during the interaction between complex NoONs such as the human body in the walking rehabilitation of the elderly (Almurad et al., 2017; Almurad et al., 2018), in dyadic communication (Abney et al., 2014), in motor control (Delignières et al., 2004; Coey et al., 2016; Delignières et al., 2016) and in interpersonal coordination (Marmelat and Delignières, 2012; Fine et al., 2015), to name a few.

It is not just in physiologic time series that fractal statistics appear, in fact, the fractal structure of physical phenomena began to be noticed shortly after Mandelbrot (Mandelbrot, 1977) introduced the fractal concept into the lexicon of science. Its utility in understanding medical phenomena took somewhat longer but it was applied to determining the spatial structure the tree-like branchings of the human lung, arterial and venous systems and other ramified structures (West and Goldberger, 1987; Liebovitch, 1998; Meakin, 1998).

### 2.2 The fractal-paradigm

The term fractal-paradigm is of fairly recent vintage and we take its use to be a consequence of the finding that naturally occurring complex phenomena have inverse power law (IPL) PDFs and consequently have fractal statistics. This empirical finding coupled to the fact that the theoretical scaling-PDF given below is the solution to a fractal kinetic equation (Sachev and Zaslavsky, 1997; Zaslavsky, 2002) entails that the nearly universal finding that natural complex penomena have fractal dimensions became a paradigm for successful science. Fractal time series exhibit self-similarity indicating that their statistical properties are invariant under different scales of observation in time (Feder, 1988). Consequently, the variability of fractal statistical fluctuations examined are the same at each level of magnification. Being fractal implies that an appparently continuous time trace such as an ECG or an EEG is actually controlled by a set of discrete statistical events generically called CETS as we have shown elsewhere (Mahmoodi et al., 2023a; Mahmoodi et al., 2023b; West et al., 2023) and is briefly reviewed herein.

We employ a working definiton of complexity, one sufficiently general to describe the generic features of physiological time series. Li (Li, 2010) points out in his excellent review article that a fractal time series can be chacterized by a fractal dimension D which is a measure of how completely the time series fills phase space and consequently provides a measure of the complexity of a CETS. Consider a typical signal generated by an *j* − ON depicted by *X*_*j*_(*t*) where *j* = 1, 2, ., which scales with an index *δ*_*j*_ if the time *t* when multiplied by a constant *λ* yields the homogeneous scaling relation $X_{j}\left(\lambda t\right)≔\lambda^{\delta_{j}}X_{j}\left(t\right)$ .The scaling relation is interpreted not in terms of the dynamic variable itself but rather in terms of the scaling-PDF such that in phase space the PDF takes the form: $P\left(x_{j},t\right)=\frac{1}{t^{\delta_{j}}}F\left(\frac{x_{j}}{t^{\delta_{j}}}\right)$ which is the renormalization group solution to the FKE as explained in SM #1. The scaling index is related to a class of fractal time series such that the fractal dimension is given by (Feder, 1988; Li, 2010; West et al., 2023): $D_{j}=2-\delta_{j}$ .Here *δ*_*j*_ is the scaling index for the CETS labeled *j* whether theoretical or empirical and when the time series is monofractal the scaling index is constant but when the time series is multifractal the scaling index becomes time dependent *δ*_*j*_(*t*) as does the MFD $D_{j}\left(t\right)$ . (see SM #1 & #3).

### 2.3 Renewal and crucal events

The theory of REs has innumerable application across a wide swath of disciplines including but not limited to actuaral science, epidemiology, operations research and reliability engineering. Herein we are interested in a class of RETS given by CETS used in the study of complex dynamic networks, such as the ONs found in the bodies of all cognitive living creatures, e.g., birds, mammals, etc., and whose nonlinear dynamics exhibit spontaneous temporal phase transitions, see discussion of SOTC (Mahmoodi et al., 2018). REs occur randomly and independently of each other having no memory of the past, whereas CEs occur randomly and independently of each other at critical points of a network’s dynamics, but have a counter-intuitive long-lasting impact on the future dynamics, even though the time intervals between succesive CE are statistically independent (see SM # 3). This counter-intuitive result was verified using the concept of “aging” where an aged survival probability generated by a CETS is shuffled and seen to have the same survival probabity as the original CETS because the time intervals between successive events are statistically independent of one another, whereas applying the aging test to FBM yields an survival probability with *δ* = 0.5. Thus, the CETS would be classified as 1/f-variability whereas FBM would be 1/f-noise, see (West and Grigolini, 2021) for a detailed discussion of this difference in 1/f-variability.

Thus, CEs are always REs, but not all REs are CEs, for example, a Poisson process is renewal and consists of a sequence of REs in a RETS that is not a CETS. A similar observation can be made for a random walk realization with *δ* = 0.5 that being simple Brownian motion with *δ* = 1/2 which is an RE sequence (see RM #3). This proved to be very important in the discussion of living networks in general and medicine in particular. The role CETS play in the communication among physiologic ONs such as the brain, heart and lungs, which, like most other biological networks, were conjectured to be on the verge of criticality (Mora and Bialek, 2011); a conjecture supported by the fractal statistics of the ON time series (Mahmoodi et al., 2018). Brain waves are rhythmic patterns of electrical activity in the brain that reflect different states of consciousness, such as wakefulness, sleep, or meditation and are typically measured using a many-channeled electroencephalogram (EEG).

## 3 Empirical findings

The basis for the empirical CS formulated here is depicted in the figure where the left panel displays 10 seconds of simultaneously recorded time series placed next to the appropriate ON drawing and the bi-directional interactions among the ONs are indicated by arrows for each ON thereby showing how different the time scales and apparent statistics are for each of the three. The typical EEG-channel for the brain is seen to resemble nothing so much as a totally random process, that of the heart could be a two-state periodic process, and the slow wave of regular breathing could be mistaken for a nonlinear wave. What is clear from this comparison of the time series is that they have nothing suggesting any of the known forms of synchronization reviewed in Section 1 controlling their unique shapes.

The raw time series for the three kinds of ONs certainly do not appear to have anything in common. Yet when the data is processed using diffusion entropy analysis (DEA) (West and Grigolini, 2021) (see also SM # 2) and their scaling statistics are revealed using DEA (see RM # 2) to produce the MFD related to their scaling indices *D*_*j*_(*t*) = 2 − *δ*_*j*_(*t*), *j* = 1, 2, ., 66, as depicted in the right-hand panel. Thus, the CS concept is vindicated by these three simultaneously recorded datasets and the coordinated quasi-periodic scaling of their respective MFD over time.

Each of the 66 channels in the figure are treated as the source of a distinct ON signal having different fractal time series with a unique MFD determined by the time-dependent scaling parameter *δ*_*j*_(*t*), *j* = 1, 2, ., 66, over a 15 min time interval. The black line segment is the average over the 64 channels of the EEG MFD fractal time series (the 64 light gray line segments in the background are the EEG channel MFDs in the brain), the red line segment is the MFD of the respiration fractal time series and the blue line segment is the MFD of the electrocardiogram (ECG) fractal time series. The quasi-periodic behavior of all 66 time series indicates the phenomenon of complexity synchronization for the three very different types of signals (Mahmoodi et al., 2023a; Mahmoodi et al., 2023b; West et al., 2023)].

## 4 Discussion and conclusions

An explicit assumption was made at the outset of this presentation having to do with the ubiquity of fractal time series being generated by ONs and their resulting multifractal nature. We now raise this explicit assumption to the level of a hypothesis since the processed datasets in the figure support the formulation of the existence of a *Law of Multifractal Dimension Synchronization* (*LMFDS*) which entails a locked-in regularity of the dynamics of the MFD time series of the interacting triad of ONs in this NoONs. In the particular case of this triad of empirical ON time series we observe that each channel of the EEG is a multifractal time series whose 1/*f* ^*β*^-variability (*β* quantifying its complexity) is the same in all cortical structures and this aligns with the observation of Buzsáki concerning brains for a variety of mammalian species (Buzsáki, 2006). The fact that the EEG channels also carry information from and deliver information to the ECG and respiratory networks, information that necessarily changes in time, thereby requiring these very different ONs to share a common language is also an empirical observation supporting the hypothetical *LMFDS*.

Buzsáki (Buzsáki, 2006) also noted that neuroscience was awaiting a breakthrough of the kind recently made in statistical physics wherein a network’s SOC (Bak, 1996) spontaneously generates structural complexity by means of the criticality of a network’s dynamics which obeys universal laws of emergence that are independent of the micro-dynamics. The newly identified mechanism of spontaneous temporal complexity given by SOTC (Mahmoodi et al., 2018) can explain the generation, transport, and exchange of information within and among the various parts of an NoONs devoted to distinctly different functions. This result is summarized by the *LMFDS* being extended beyond the physiologic datasets used herein to support its veracity which in addition has strong empirical arguments for its support [2, ? 38, 54] outside of physiology yjereby making the conjecture for the extended application-domain of the *LMFDS* hypothesis reasonable.
