# Supplementary material for: Complexity synchronization in living matter: a mini review
Source: Front Netw Physiol. 2024 May 20;4:1379892. doi: 10.3389/fnetp.2024.1379892 (PMC11145412; doi:10.3389/fnetp.2024.1379892)
Supplement: Supplementary file 1 [file DataSheet1.PDF]

Supplimentary Material
for
"Complexity Synchronization in Living Matter"

Bruce J. West*
Center for Nonlinear Sceince, UNT, Denton, TX
and
Department for research and Innovation, NCSU, Raleigh, NC
*brucejwest213@gmail.com

1 Fractal-paradigm and the fractional calculus

One aspect of the fractal paradigm concerns itself with the 1/f-behavior of the self-similar statistics of a fractal time series in which this 1/f-variability is because the underlying process does not vary in "chronological time" but in an "intrinsic time" that is a form of fractal time [6]. The fractal time has to do with the fact that the events are crucial and therefore in addition to their being renewal the time interval statistics are IPL with an IPL index within the interval $1 < \mu < 3$. The fractal time suggests that the description of the evolution of the PDF is determined by the fractional probability calculus [7].

A sketch of the formal solution to a Fractional Kinetic Equation (FKE) for the scaling PDF is presented using renormalization group theory. The equation to be solved was first derived using chaos theory and the fractional calculus by Zaslavsky [**?**] and subsequently rederived [8] using the continuous time random walk model of Montroll and Weiss. Consequently, the FKE describing the space-time evolution for the PDF is determined to be:

$$D_t^{\alpha}\left[P\left(x,t\right)\right] = K_{\beta} D_{|x|}^{\beta}\left[P(x,t)\right], \tag{1}$$

where the Caputo fractional derivative in time is operating on the left side of the equation and the Riesz-Feller fractional derivative in space is operating on the right side of the equation. The FKE is solved as an initial value problem for the FPD, where $P_0(x) = P(x, t = 0)$.

Zaslavsky et al. [7, 12] applied the renormalization group transformation R to the network dynamics such that the scaling properties of the incremental changes are

$$R : \delta x = \lambda_x \delta x \ ; \ R : \delta t = \lambda_T \delta t, \tag{2}$$

which apply, after some averaging, to a restricted space-time domain and the scaling

parameters are $(\lambda_x; \lambda_T)$. They continue with the observation that the FKE of Eq.(1) is invariant under the renormalization group transformation:

$$R : D_t^{\alpha} [P(x,t)] = K_{\beta} R : D_{|x|}^{\beta} [P(x,t)], \tag{3}$$

which implies that the transformed fractional equation satisfies the scaling behavior:

$$\lambda_T^{\alpha} D_t^{\alpha} [P(x,t)] = K_{\beta} \lambda_x^{\beta} D_{|x|}^{\beta} [P(x,t)]. \tag{4}$$

The lowest-order renormalization group solution is given by equating the renormalization parameters raised to their respective powers: $\lambda_T^{\alpha} = \lambda_x^{\alpha}$. The solution to the renormalized FKE is given in terms of the Fourier transform of the PDF, which is the characteristic function $\phi(t)$, expressed in terms of the Mittag-Leffler function $E_{\beta}(.)$ to be:

$$\phi(t) = E_{\beta}\left(-K_{\beta} |k|^{\beta} t^{\alpha}\right). \tag{5}$$

Consequently, taking the inverse Fourier transform of this characteristic function and expressing the Mittag-Leffler function as an infinte series, after some algebra [12, 9], results in the scaling solution for the PDF:

$$P\left(x\lambda_T^{\alpha/\beta}, t\right) = P(x,t)/\lambda_T^{\alpha/\beta}. \tag{6}$$

Selecting the ratio of the scaling parameters to be the exponent of the remaining scaling parameter:

$$\delta = \alpha/\beta, \tag{7}$$

and choosing the time scale of interest to reduce the dependence of the PDF on the left side of the equality in Eq.(SM1.6) to be dependent on the single scaled variable x/t^{δ} using Eq.(7):

$$\lambda_T = 1/t, \tag{8}$$

enables us to rewrite Eq.(6) in the form of the scaling PDF given in Section 2.2. Thus, the solution to the FKE yields the scaling PDF used, along with the DEA data processing technique, to interpret the datasets from the brain, heart and lungs in the text as CETS with synchronized levels of complexity as measured by their respective multifractal dimensions.

2 Diffusion Entropy Analysis (DEA)

In this section we indicate how to determine the empirical IPL scaling parameter's consistency with the theory presented in the previous section. This is accomplished using the DEA to process the recorded time series generated by the members of the triad of ONs consisting of the heart, lungs and brain. The main text contains the results of applying this method to the 66 ONs of the triad. The sequential steps in the DEA technique referenced to Figure 1 are:

1.)Project the raw data of each channel onto the interval [0,1] by normalizing each time series by the total time interval of the dataset thereby enabling the processing of each time series to be directly compared.

2.) Divide the normalized data profile into parallel stripes of size of 0.01 (panel (a), ECG data).

3.) Extract events by defining them as unit amplitude pulses if the signal at that time is in a different stripe with respect to its previous value (panel (b)) and zero if it remains in the same stripe.

4.) Create a diffusion trajectory (panel (c)) using the time series of the extracted events from step 3.

5.) Determine the statistics of a single diffusion trajectory by selecting a window size w and partitioning the diffusion trajectory into many pieces, each starting from an event.

6.) Initiating all the trajectories from an event enables their being shifted to start from a common origin (panel (d)).

7.) Finally, we evaluate the ensemble distribution of histograms at a given time (panel (e)) since the events are statistically independent.

To make the statistics of the single time series diffusion trajectory correspond to that done in the DEA processing of the data we pick a window $w = t$ starting from a common origin event (panel d) and evaluate the distribution of trajectories at time t (panel e). Denoting the PDF for different window sizes as *P(x,w)* we can define the SW-entropy as:

$$S(t) = -\int dx P(x,w) \log_2 P(x,w). \tag{9}$$

Assuming that *P(x,w)* is the PDF corresponding to window size w we can define diffusion entropy using the SW-entropy as being the information contained in the time series. Using the scaling PDF, without knowing the $F(.)$ PDF, the deviation of the SW-entropy from its reference state defined by the unknown function is:

$$\Delta S(w) = S(w) - S_{ref} = \delta \log_2 w. \tag{10}$$

Consequently, if a graph of the SW-entropy for an empirical process versus the logarithm of the time yields a straight line we interpret the positive slope to be the scaling index δ as done in the main text.

Healthy living network host healthy ONs by having equal strength in transferring information among ONs in a NoONs but give rise to time series whose mutifractal fluctuations contain control information that guides both the internal behavior in intra-ON and external information exchange during inter-ONs within the NoONs. In fact, the health of the human body is determined by the multifractal dimensions (MFDs) $D_j(t)$ or equivalently by the scaling indices $\delta_j(t)$ due to the generic relation between the two quantities: $D_j(t) =$

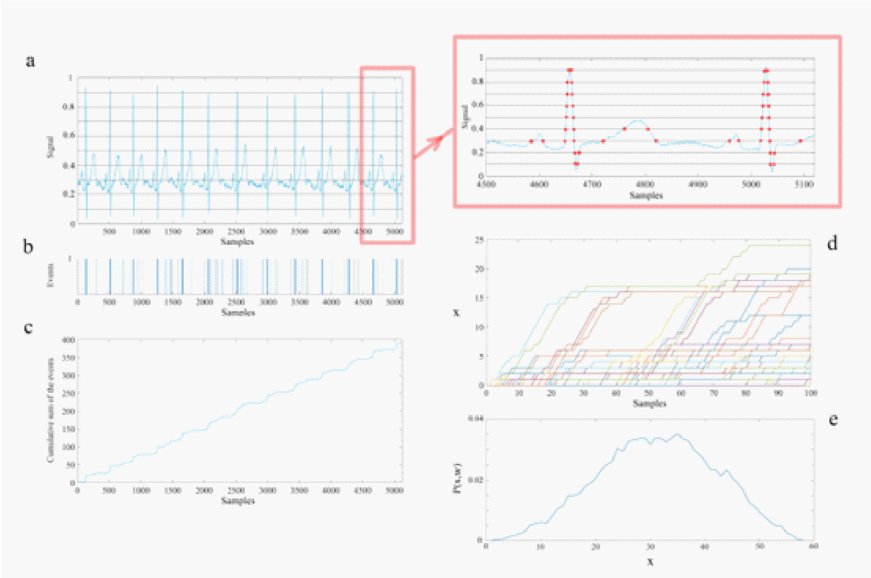

Figure 1: A schematic of the steps for the processing of time series using the technique Diffusion Entropy Analysis. Panel a): The solid curve is the heart rate signal projected onto the interval [0, 1] divided by the stripe size of 0.1, which is magnified in the inset. Note that sharply peaked features in the ECG have a cluster of events in (b), whereas a sloping feature has well-spaced events, see the inset for a visual verification of this explanation. The horizontal lines define the stripes. Panel b): The events (represented as distinct separated unit positive amplitude pulses) are extracted from the passage of the continuous time trace of the ECG from one stripe to another. Panel c): The diffusion trajectory made by the cumulative summation of the events of panel (b). The vertical lines show a selected set of windows with sizes 100 that sliced the diffusion trajectory. Panel d): The partitioned trajectories of panel (c) shifted to initiate each trajectory from a common origin and terminate each after a time w=t, the length of the window. Panel e): The histogram of the position of the trajectories at the end of the windows (to create this histogram 60 sec of data and stripe width of 0.01 which were the DEA parameters used in the data processing.) Taken from [11] with permission.

$2 - \delta_j(t)$. The scaling index has the ideal value of one as the condition for health of the human body. It is a singular condition corresponding to the largest possible scaling and has been used in the analysis of time series as a diagnostic indicator separating patients who have congestive heart failure from those that are healthy[1, 2].

3 Crucial Events and CS

Empirically the phenomenon of CS is based on time series consisting of random discrete events with renewal statistics which enable two such interacting ONs to detect and quantify the complexity of each ON and to minimize the difference in their complexity so as to become synchronized. The quantification of complexity is given by the MFD which becomes equal in each ON time series even though they may be operating on largely different time scales, such as the brain, heart and lungs. The discrete events in such time series have been named crucial because they emerge from the sub-dynamics to determine the efficiency of information exchange among the ONs.

A CETS is generated by a RETS with an IPL waiting-time PDF $\psi(\tau) \propto \tau^{-\mu}$ having an IPL index in the domain $1 < \mu < 3$. Asymptotically, the generated CETS describes an ergodic process for the IPL index μ in the range $2 < \mu < 3$ with a finite average waiting time. Ergodic is the technical name for statistical processes for which averages taken over long time series are equal to those taken over PDFs. The understanding of complexity in many-body physics is formally understood by assuming the 'ergodic hypothesis' dating back over a century to the time of Boltzmann

The unknown function $F(\cdot)$ of the scaled variable $y = x/t^{\delta}$ in a scaled PDF and is unknown in general but is well known for certain values of the scaling parameter. This generic form of the solution is obtained from an equation of evolution for the PDF that has an $\alpha-$order derivative in time and a $\beta-$order derivative in space such that the solution to the fractional-order diffusion equation can be solved using renormalization group theory as shown in SM #1 to obtain the scaling PDF with the scaling index $\delta = \alpha/\beta$ [sm8]. The ordinary diffusion equation has the integer-order indices $\alpha = 1$ and $\beta = 2$ yielding the well-known scaling index $\delta = 1/2$ in which case the unknown function $F(y)$ becomes a Gaussian with the scaled variable $y = x/\sqrt{t}$. Note that the fractal dimension for this process is obtained from $\mathcal{D} = 2 - \delta = 1.5$ which is therefore a monofractal process that is completely random, which is to say the diffusion process has no memory. Consequently, scaling PDFs with $\delta \neq 1/2$ are anomolous in that their fractal dimension is $D > 1.5$ indicating that $\alpha < 1$ with $1 < \beta \leq 2$.

If the anomoly has a completely temporal origin we have for the spatial derivative index $\beta = 2$ for a spatially homogeneous process and $\delta < 1/2$ thereby producing the fractal dimension $\mathcal{D} > 1.5$ indicating the existence of a memory-like process. The more the scaling index deviates from 1/2 the more the fractal dimension deviates from 1.5 and the stronger the anomoly. If the time deviation

is produced by a fractal time series with an IPL waiting-time PDF which when these time intervals are statistically independent of one another constitues a *renewal* time series. The renewal time series is ergodic for $2 < \mu < 3$ with a finite average waiting time and is non-ergodic for $1 < \mu < 2$ with a diverging average waiting time.

Suppose the trajectory $X(t)$ crosses a known level at a specific time and we want to know how long we must wait to recross that same level. Given that the waiting-time PDF has the IPL form we denote the generic IPL index for the waiting-time PDF μ by the symbol $\mu_{\mathcal{D}}$. From the other parameter relations in Table 1, it is clear that it is possible to prove that the IPL index is equal to the fractal dimension, so that we obtain [4, 11]:

$$\mu_{\mathcal{D}} = 2 - \delta, \tag{11}$$

which is possible to establish using the probability of crossing and recrossing any fixed value of the diffusion trajectory which has been shown to be a renewal process [sm11] and is consequenly a CETS.

The power spectral density $S_p(f)$ (PSD) for a CETS is also IPL in terms of the frequency f:

$$S_p(f) \propto f^{-\beta}, \tag{12}$$

indicating $1/f-$variability. This IPL PSD is the Fourier transform of a slowly decaying autocorrelation function indicative of a fractal time series having global or local self-similarity. The IPL indices μ and β are interrelated for the class of fractal time series of interest in medicine such that the fractal dimension given by Eq.(11) is related to the IPL PDF index defined by Eq.(12), and the IPL index for the PSD is [10]:

$$\beta = 3 - \mu. \tag{13}$$

Thus, true $1/f-$noise at $\beta = 1$ arises only at the boundary $\mu = 2$ between the ergodic and non-ergodic variability domains.

Table 1 is copied from West et al. [11] and provides easy reference to all the derived relations among the IPL scaling parameters for the PSD index β, the IPL waiting-time PDF index μ and the scaling parameter δ for the scaled variable $X(t)$. The channel index j is omitted from the tabled indices since it does not add any additional information.

**Table 1**	scaled functions	parameter relations	parameter range	
waiting-time PDF	$\psi(\tau) \propto \tau^{-\mu}$		$1 < \mu < 3$	
power spectrum	$S_p(f) \propto f^{-\beta}$	$\mu = 3 - \beta$		
scaled variable	$X(t) \propto t^{\delta}$	$\mu = 1 + \delta$	$1 < \mu \leq 2$	non-ergodic
		$\mu = 1 + 1/\delta$	$2 \leq \mu < 3$	ergodic
		$\delta = 1/2$	$\mu \geq 3$	ergodic

In this table we record the scaling index δ from the homogeneous scaling relation for the scaled variable $X(t)$, relating it to the IPL power spectrum index β through the waiting-time PDF $\psi(\tau)$ IPL index μ. The value $\mu = 2$ is the boundary between the underlying process having a finite ($\mu > 2$) or an

infinite ($\mu < 2$) average waiting time and is also the point at which $\beta = 1$ where the process is that of true $1/f-$noise. Consequently, β and μ are interchangeable measures of complexity. For an ergodic time series such as that determined by the waiting-time inverse power-law index μ increases with decreasing scaling index δ and the fractal dimension increases. Adapted from [5] with permission.

References

[1] G. Bohara, D. Lambert, B. J. West, and P. Grigolini, "Crucial events, randomness, and multifractality," Physical Review E 96, 062216 (2017).

[2] G. Bohara, B. J. West, and P. Grigolini, "Bridging waves and crucial events in the dynamics of the brain," *Frontier in Physiology* **9**, 1174 (2018).

[3] R Failla, P Grigolini, M Ignaccolo, and A Schwettman, "Random growth of interfaces as a subordination process", *Phys. Rev. E* **70**, 010101 (R) (2004).

[4] FJ Feder, *Fractals*, Plenum Press: New York, NY (1988).

[5] K Mahmoodi, SE Kerick, P Grigolini, PJ Franaszczuk, and BJ West, "Complexity synchronization: a measure of interaction between the brain, heart and lungs", *Sci. Rep.* **13**, 11433 (2023).

[6] B.B. Mandelbrot, "1/f noises and the infrared catastrophe", *IEEE Comm. Conv.*, Boulder, CO (1965).

[7] A. I. Saichev and G. M. Zaslavsky, "Fractional kinetic equation: solutions and applications," *Chaos* 7, 753-764 (1997).

[8] B.J. West and P. Grigolini, *Complex Webs: Anticipating the Improbable* (Cambridge University Press, 2011).

[9] B. J. West, *Fractional Calculus View of Complexity: Tomorrow's Science*, CRC Press, (2016).

[10] BJWest and P. Grigolini, *Crucial Events: Why are Catastrophies Never Expected?*, World Scientific, Singapore (2021).

[11] BJ West, P Grigolini, SE Kerick, PJ Franaszczuk and K Mahmoodi, "Complexity Synchronization of Organ Networks", *Entropy* **25**, 1393-1412 (2023). https://doi.org/10.3390/e25101393.

[12] G. M. Zaslavsky, "Chaos, fractional kinetics, and anomalous transport," *Phys. Rept.* **371**, 461 (2002).
